# Long-Term Use of Tedizolid in Osteoarticular Infections: Benefits among Oxazolidinone Drugs

**DOI:** 10.3390/antibiotics10010053

**Published:** 2021-01-08

**Authors:** Eva Benavent, Laura Morata, Francesc Escrihuela-Vidal, Esteban Alberto Reynaga, Laura Soldevila, Laia Albiach, Maria Luisa Pedro-Botet, Ariadna Padullés, Alex Soriano, Oscar Murillo

**Affiliations:** 1 Infectious Diseases Department, Hospital Universitari de Bellvitge—IDIBELL, L’Hospitalet de Llobregat, University of Barcelona, 08907 Barcelona, Spain; eva.benavent@bellvitgehospital.cat (E.B.); fescrihuela@bellvitgehospital.cat (F.E.-V.); laura.soldevila@bellvitgehospital.cat (L.S.); 2 Bone and Joint Infection Study Group of the Spanish Society of Infectious Diseases and Clinical Microbiology (GEIO-SEIMC), 28003 Madrid, Spain; lmorata@clinic.cat (L.M.); asoriano@clinic.cat (A.S.); 3 Infectious Diseases Department, Hospital Clínic-IDIBAPS, University of Barcelona, 08036 Barcelona, Spain; lalbiach@clinic.cat; 4 Spanish Network for Research in Infectious Diseases (REIPI RD16/0016/0003), Instituto de Salud Carlos III, 28029 Madrid, Spain; 5 Department of Infectious Diseases, Hospital Germans Trias i Pujol, 08916 Barcelona, Spain; ereynaga@hotmail.com (E.A.R.); mlpbotet.germanstrias@gencat.cat (M.L.P.-B.); 6 Department of Pharmacy, Hospital Universitari de Bellvitge—IDIBELL, L’Hospitalet de Llobregat, University of Barcelona, 08907 Barcelona, Spain; apadulles@bellvitgehospital.cat

**Keywords:** tedizolid, oxazolidinones, osteoarticular infections, diabetic foot infections, drug-drug interaction

## Abstract

Background: To evaluate the efficacy and safety of long-term use of tedizolid in osteoarticular infections. Methods: Multicentric retrospective study (January 2017–March 2019) of osteoarticular infection cases treated with tedizolid. Failure: clinical worsening despite antibiotic treatment or the need of suppressive treatment. Results: Cases (*n* = 51; 59% women, mean age of 65 years) included osteoarthritis (*n* = 27, 53%), prosthetic joint infection (*n* = 17, 33.3%), and diabetic foot infections (*n* = 9, 18%); where, 59% were orthopedic device-related. Most frequent isolates were Staphylococcus spp. (65%, *n* = 47; S. aureus, 48%). Reasons for choosing tedizolid were potential drug-drug interaction (63%) and cytopenia (55%); median treatment duration was 29 days (interquartile range -IQR- 15–44), 24% received rifampicin (600 mg once daily) concomitantly, and adverse events were scarce (*n* = 3). Hemoglobin and platelet count stayed stable throughout treatment (from 108.6 g/L to 116.3 g/L, *p* = 0.079; and 240 × 10^9^/L to 239 × 10^9^/L, *p* = 0.942, respectively), also in the subgroup of cases with cytopenia. Among device-related infections, 33% were managed with implant retention. Median follow-up was 630 days and overall cure rate 83%; among failures (*n* = 8), 63% were device-related infections. Conclusions: Long-term use of tedizolid was effective, showing a better safety profile with less myelotoxicity and lower drug-drug interaction than linezolid. Confirmation of these advantages could make tedizolid the oxazolidinone of choice for most of osteoarticular infections.

## 1. Introduction

Oxazolidinones are a young family of antibiotics that have a wide action against Gram-positive bacteria and include the first designed linezolid and the recent tedizolid. In comparison with the former, tedizolid has shown a higher in vitro activity against some microorganisms (four- to eight-fold lower minimum inhibitory concentration (MIC) values), and its pharmacokinetic/pharmacodynamics parameters allow the once-daily administration, which may improve treatment adherence. Also, tedizolid at approved doses seems to provide a better safety profile and less adverse events than linezolid, especially in relation with myelotoxicity and drug–drug interaction [1,2,3].

Tedizolid is currently only approved for acute bacterial skin and skin structure infections (ABSSSIs), not including diabetic foot infections [4,5]. However, it seems reasonable that tedizolid can be used in other clinical settings where linezolid has played a relevant role. In this sense, difficult-to-treat osteoarticular infections mainly due to staphylococci constitute a notable scenario where linezolid has provided good efficacy [6,7,8]. However, adherence to linezolid treatment may involve some difficulties such as (i) the appearance of myelotoxicity at two to four weeks of treatment, since osteoarticular infections usually require longer treatments [8,9]; (ii) the decrease in linezolid serum levels when combined with rifampicin, an anti-staphylococcal agent widely used in device-related infections [10,11,12,13]; and (iii) the risk of serotonin syndrome when administered concomitantly with antidepressants broadly used nowadays [14,15]. 

Despite the potential advantages of tedizolid in osteoarticular infections (higher microbiological activity, advantageous pharmacokinetic/pharmacodynamics parameters, lower myelotoxicity, and drug–drug interactions) [1,2,3], clinical data on long-term treatment are scarce [16]. Knowledge is limited to a few experimental studies [17,18,19], case reports [20] and recently some case series in which tedizolid is prescribed for different indication including osteoarticular infections [21,22]. Thus, in the present study, we intend to describe our multicenter experience within the Spanish Network for Research in Infectious Diseases (REIPI) with long-term use of tedizolid in a cohort of patients with osteoarticular and diabetic foot infections and focused on the efficacy and safety in monotherapy or combination.

## 2. Results

A total of 51 cases were included in our study. Mean age was 64.8 ± 14.3 and 59% (*n* = 30) were women. Median Charlson Index adjusted by age was 4 (IQR 3–7). Obesity was present in 33% (*n* = 15) of the cases; other frequent comorbidities were diabetes mellitus (*n* = 21, 41%), chronic renal disease (*n* = 16, 31%), malignancies (*n* = 7, 14%), and chronic anemia (*n* = 4, 8%).

There were 53% (*n* = 27) diagnosis of osteoarthritis (*n* = 17 cases of peripheral osteomyelitis, *n* = 6 septic arthritis and *n* = 4 vertebral osteomyelitis; of which 1 case presented simultaneously with septic arthritis of the ankle and osteomyelitis of the tibia), 33% (*n* = 17) cases had a prosthetic joint infection (*n* = 8 post-surgical acute, *n* = 8 chronic and 1 case with intraoperative positive cultures), and there were 18% (*n* = 9) cases of diabetic foot infection, one of them presenting also with vertebral osteomyelitis. Thirty cases (59%) were orthopedic device-related infections. Only two cases (4%) had bacteremia (both due to methicillin-susceptible *Staphylococcus aureus*).

Microorganisms responsible for osteoarticular infections were identified in all but two cases. There were 20 cases (39%) of polymicrobial etiology, 11 of them at the expense of different Gram-positive isolates that were all treated with tedizolid, and the remaining cases were mixed with Gram-positive and Gram-negative microorganisms. All Gram-positive microorganisms involved are presented in Table 1.

Tedizolid was administered at a dosage of 200 mg per day orally for a median of 29 days (IQR 15–44); 63% of the cases (*n* = 32) received tedizolid for more than 21 days, and in 70% of the cases (*n* = 36) time under tedizolid treatment represented more than 50% of the whole antibiotic treatment duration. Causes for prescription of tedizolid are presented in Table 2 (in 14 cases there was more than one reason); most common reason for initiate tedizolid was the potential interaction between baseline treatment and linezolid (65%), followed by the presence of anemia and/or thrombocytopenia (37%) and toxicity caused by a previous antibiotic (16%).

Tedizolid was mainly administered as part of sequential switching therapy (*n* = 47, 92%; 3 cases as salvage therapy after failure), and only in 4 cases (8%) was the initial treatment. Tedizolid was given in monotherapy (*n* = 27, 53%) and in combination (*n* = 24, 47%) almost in similar proportion; among combination therapy, in half of the cases (*n* = 12) tedizolid was combined with rifampicin (600 mg once daily) representing 25% of all staphylococci infections. Less usual tedizolid combinations were used to treat polymicrobial infections with participation of Gram-negative bacteria; among them the most frequent drugs were quinolones (*n* = 7, 14%) and carbapenems (*n* = 4, 5%).

**Figure 1:**
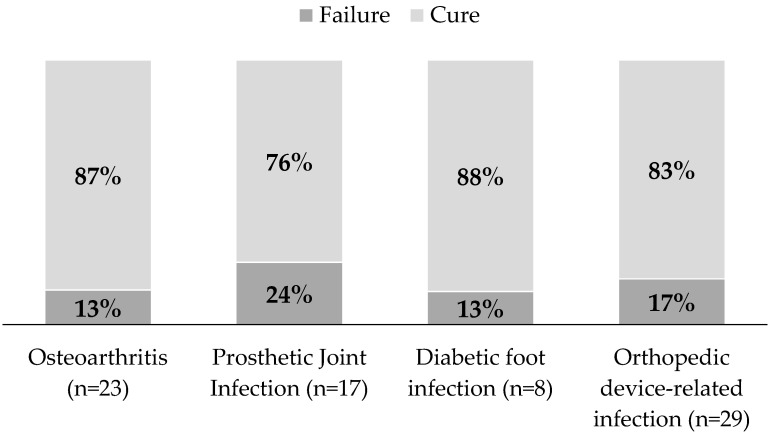
Failure rates among different types of osteoarticular infection.

Beside therapy with tedizolid, most of the cases were managed surgically (*n* = 41, 80%); among device-related infections, implants were removed in 57% cases. Among cases with an evaluable outcome (*n* = 48; median of follow-up 630 days, IQR 269–818), the overall cure rate was 83% and 8 cases (17%) failed. There were 3 deaths, all of them non-related neither with the infection nor with tedizolid therapy, in which the outcome could not be evaluated due to short follow-up after treatment. The cure and failure rates among each type of osteoarticular infection are presented in Figure 1. Among failures, 4 of them were prosthetic joint infections (3 of them were put on suppressive antibiotic treatment and the remaining one underwent further surgery to cure the infection), 3 cases of osteoarthritis (one case was a device-related infection managed with implant removal), and a case of diabetic foot infection. Among staphylococci infections, there was no difference in outcome when tedizolid was used in monotherapy vs. combination with rifampicin (failure rate of 21% vs. 0%, respectively, *p* = 0.118).

Therapy with tedizolid was well tolerated; the only adverse effect observed was gastrointestinal disturbances in three cases (6%; nausea and occasional vomiting), but in any case, treatment was withdrawn.

**Figure 2:**
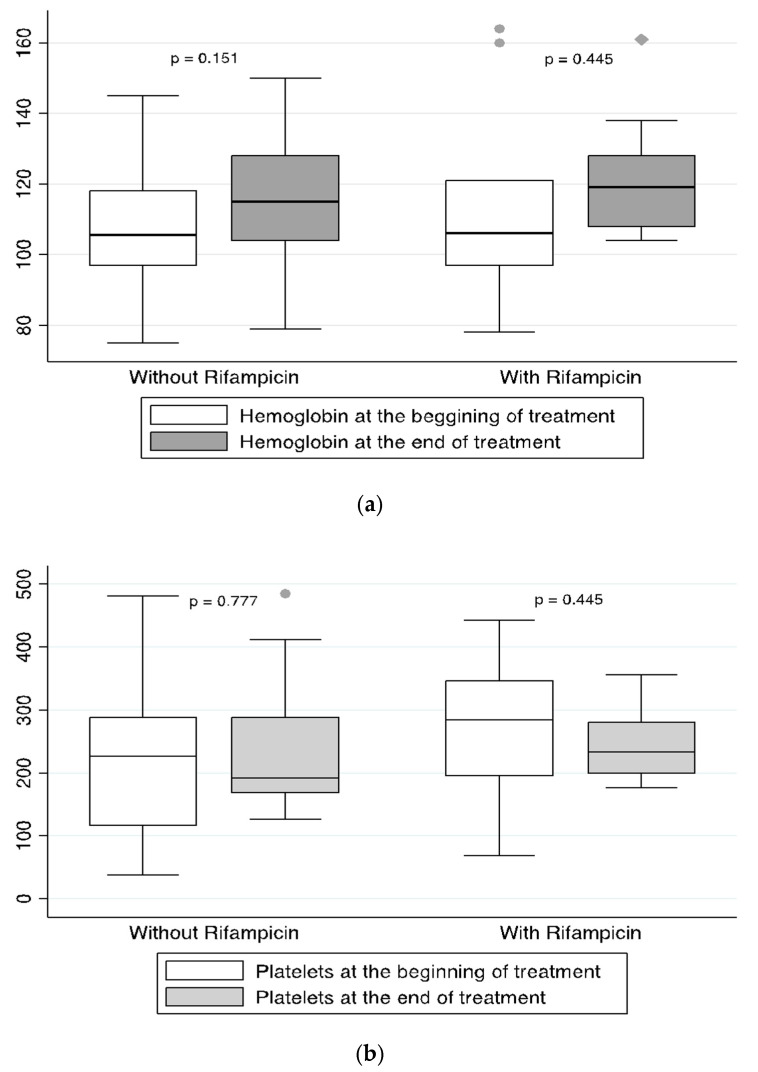
Mean comparison of hemoglobin values and platelet count at the beginning and the end of treatment for those cases receiving Tedizolid in monotherapy (**a**) or combination with Rifampicin (**b**).

There was no worsening on the hemoglobin or platelet counts in the blood tests between the beginning and the end of treatment with tedizolid neither in the group of patients with cytopenia nor in those without (Table 3) or those where treatment was prolonged for more than 21 days. In three cases, linezolid was switched to tedizolid because myelotoxicity of the former and patients completed treatment without additional worsening. The use of rifampicin in combination with tedizolid did not produce significant differences in the final levels of hemoglobin or platelets in comparison with tedizolid in monotherapy (Figure 2).

Finally, among cases treated with tedizolid because of potential interaction between linezolid and baseline treatment (*n* = 33), they were treated for a median of 34 days (IQR 17–51) and we did not observe adverse events (i.e., serotonin syndrome) or alteration of basal disease (i.e., depressive syndrome) either during therapy with tedizolid or after it was stopped. 

## 3. Discussion

Tedizolid is recommended for the treatment of ABSSSIs for six days and still, there is limited information in other settings or prolonged treatments. In the present study, we provide data about efficacy, the safety of long-term use (median of 29 days), and benefits of tedizolid in a large cohort of patients with osteoarticular and diabetic foot infections. 

Linezolid, the first approved oxazolidinone drug, has provided good outcomes in osteoarticular infections [6,7,8,12]; however, there are still some concerns for its long-term use regarding adverse events and drug–drug interactions. Comparing with linezolid, tedizolid shows a better microbiological activity and more favorable pharmacokinetic/pharmacodynamics parameters when used at the recommended dose of 200 mg daily. In our experience, tedizolid both in monotherapy or combination provide good efficacy in this field (cure rate 83%), comparable to that of linezolid. These results are difficult to compare with previous work, because to our knowledge, there are no studies focused on tedizolid efficacy in diabetic foot infections and only a recent experience of tedizolid for more than six days in different types of infections, including some osteoarticular infection cases [22]. Thus, larger experience in this setting is needed, but our results seem to be in the line of considering tedizolid a good therapeutic alternative. 

Among adverse events observed when using linezolid, anemia and/or thrombocytopenia is common when its use is prolonged beyond two weeks [9,12,23]. It requires monitoring patients, especially those with previous cytopenia or particular risk factors. Tedizolid can also cause dose-related myelotoxicity [16], but at a lower rate than linezolid [24]. Safety of long-term use of tedizolid was evaluated in healthy volunteers for 21 days [16], while most of the information in patients is limited to six days of treatment in ABSSSIs [4,5,21], and there is little information with longer therapies where the appearance of thrombocytopenia and anemia was observed in 7.4 and 1.2%, respectively [22]. In our experience, tedizolid was administered a median of 29 days and was well tolerated without relevant hematologic adverse events appearing or need for withdrawn, even in cases with moderate/severe cytopenia at the start of therapy or those that were switched to tedizolid after developing linezolid myelotoxicity [25].

Among the most relevant drug–drug interactions of linezolid, its use with rifampicin should be emphasized since it is a major anti-staphylococcal agent broadly used in osteoarticular infections always in combination. Previous studies confirmed the interaction between both drugs [3,13,26], as a result, serum linezolid levels decrease [10,11]. Interestingly, this effect between tedizolid and rifampicin was not found in preclinical studies [3,17]; however, well-designed clinical and pharmacokinetic studies are not available. In our experience, despite not determining the comparative serum levels of tedizolid when monotherapy or combination with rifampicin was used, we did not observe differences in the clinical outcome or the impact on hemoglobin or platelet counts in both groups. If the absence of interaction between rifampicin and tedizolid is confirmed, the latter could displace the use of linezolid in those patients who require it in a rifampicin combination.

Finally, treatment with linezolid can also be challenging when given concomitantly with monoamine oxidase inhibitors and other antidepressants due to the risk of serotonin syndrome, a rare but serious complication [14,15]. Tedizolid exhibits a weak reversible monoamine oxidase inhibition in vitro effect, so drug–drug interaction is lower [27]. The potential drug–drug interaction was the main reason (62.8%) for choosing tedizolid in our cohort, and none of the patients interrupted their baseline treatment and no adverse event was observed.

Our study has several limitations inherent to its retrospective nature, as well as the heterogeneity between the different osteoarticular infections, and the limited number of cases. As a result, the inference of our results in particular scenarios should be taken with caution while waiting for wider experience. Also, the different surgical approaches, which are a cornerstone of the treatment of osteoarticular infections, and the use of other antibiotics before tedizolid or in combination (mainly with rifampicin), may have played a role in the overall outcome. Furthermore, to assess the suitability of the rifampicintedizolid combination or the use of tedizolid concomitantly with antidepressants, further specific studies are needed. However, to our knowledge this is the first study assessing long-term use of tedizolid specifically in osteoarticular infections and carried out by specialists in the field and, therefore, the information provided in terms of efficacy and safety is of interest. 

## 4. Materials and Methods

### 4.1. Study Population and Settings

We conducted a retrospective multicenter study in three Spanish hospitals of the REIPI-GEIO Network (January 2017 to March 2019). We included adult patients attended for osteoarticular and diabetic foot infections caused by Gram-positive bacteria who had received as part of their antibiotic treatment tedizolid at a regimen dose of 200 mg daily for at least 7 days. Polymicrobial infections with the participation of Gram-negative microorganisms were also included, whenever they received tedizolid in combination for their treatment. Those cases where Gram-positive bacteria were involved after a different primary infection (superinfection) were excluded.

We aimed to evaluate the efficacy and safety of cases treated with tedizolid for a long-term period. Additionally, we aimed to evaluate the potential drug–drug interaction between tedizolid and rifampicin or antidepressants.

### 4.2. Definitions and Data Collection

Osteoarticular infections were classified into 3 groups: osteoarthritis (including cases with peripheral or vertebral osteomyelitis and septic arthritis), prosthetic joint infections, and diabetic foot infections. All cases met the main diagnostic criteria [28,29,30] and management was carried out according to the attending medical team, in all cases, antibiotic treatment was tailored by infectious diseases specialists.

Presence of anemia was classified in mild anemia when hemoglobin concentration was between 110–129 g/L for men and 110–119 g/L for women, moderate anemia when hemoglobin was below 109 g/L, and severe when it was below 80 g/L. Thrombocytopenia was considered when platelet count was below 150 × 10^9^/L. 

Demographic data and baseline characteristics were collected. The presence of depressive syndrome was specifically registered and the use of drugs with potential major interaction with oxazolidinones such as mono-amino oxidase inhibitors, selective serotonin reuptake inhibitors, opioids, and anticonvulsant drugs. The presence or absence of orthopedic devices was documented and also the need for debridement surgery and removal or exchange of orthopedic devices when necessary. Antibiotic treatment previous and concomitant with tedizolid was also recorded. Microbiologic data were obtained from intraoperative cultures, joint fluid samples, or targeted biopsies.

Written informed consent was considered not necessary for the study, as it was a retrospective analysis of our clinical practice. Data of patients were anonymized for the purposes of this analysis. Confidential information of patients was protected according the Declaration of Helsinki. This manuscript was revised for its publication by Research Ethics Committee of Bellvitge University Hospital (PR459/20). 

### 4.3. Follow Up and Outcome

To monitor possible hematologic toxicity, we documented laboratory data at the beginning of treatment with tedizolid, during treatment, and at the end of the antibiotic treatment. The patients also underwent clinical follow-up to detect the presence of other adverse events; gastrointestinal or neuropathic toxicity (optical and peripheral).

Cases were considered cured when there was no clinical evidence of infection and no other need for antibiotic or surgical treatment once treatment with tedizolid was concluded. Failure was considered when reappearance of infection signs once treatment was already concluded, in cases of none improvement despite active treatment with tedizolid, the need of suppressive antibiotic therapy to control the infection or death related with the infection.

### 4.4. Statistical Analysis

Data were analyzed with Stata 14.2 (Stata Corporation, Texas 77845, USA). Categorical variables were described by counts and percentages, while mean and standard deviation or median and interquartile range (IQR) were used to summarize continuous variables. Comparisons between groups were performed with either the chi-square test or Fisher exact test for categorical variables, and the *t*-test or Mann-Whitney U test was used for continuous variables. 

## 5. Conclusions

In conclusion, tedizolid was effective and safe providing a valid therapeutic alternative for osteoarticular infections. Its higher microbiological activity and better pharmacokinetic/pharmacodynamics parameters comparing with linezolid, allow it to be used at doses that show a better safety profile with less myelotoxicity and lower drug-drug interaction including rifampicin and antidepressants. If further studies confirm these advantages, tedizolid may become the oxazolidinone of choice in most patients with osteoarticular infections.

## Figures and Tables

**Table 1:** Gram-positive microorganisms responsible of osteoarticular infections and treated with tedizolid, from the 72 isolates in the whole cohort.

Microorganism	Total	%
*Staphylococcus spp*	47	65.3%
*Staphylococcus aureus*	21	29.2%
Methicillin resistant *S. aureus*	10	47.6%
Coagulase negative staphylococci	26	36.1%
*Staphylococcus epidermidis*	18	25%
Others	8	11.1%
Other gram positives	13	18.1%
*Corynebacterium striatum*	4	5.6%
*Enterococcus spp.* ^1^	4	5.6%
*Streptococcus spp.* ^2^	3	4.2%
*Cutibacterium acnes*	1	1.4%
*Actinotignum schaali*	1	1.4%

^1^ Enterococcus faecium *n* = 3, Enterococcus raffinosus *n* = 1. ^2^ Streptococcus pyogenes *n* = 1, Streptococcus oralis *n* = 1, Streptococcus agalactiae *n* = 1.

**Table 2:** Reasons for Tedizolid prescription.

Reasons for Tedizolid prescription	N	%
Potential interaction with Linezolid	33	64.7%
Antidepressants ^1^	26	51%
Opioids	12	23.5%
Neuroleptics	4	7.8%
Anticonvulsants	2	4%
Cytopenia	19	37.3%
Anemia	10	19.6%
Thrombocytopenia	1	2%
Both	8	15.7%
Toxicity of previous antibiotic treatment	8	15.7%
Failure of previous antibiotic treatment	3	5.9%
Other ^2^	2	3.9%

^1^ All cases were under treatment with Serotonin Reuptake Inhibitors (SRIs). ^2^ Shortage of Linezolid.

**Table 3:** Analytic values of patients under Tedizolid treatment.

Hematological Parameters	N	At the Beginning of Treatment with Tedizolid (mean, SD)	At the End of Treatment with Tedizolid (mean, SD)	*p* Value	Use of Rifampicin	Days with Tedizolid (Median, IQR)
Hemoglobin (g/L)	45	108.6 ± 20.3	116.3 ± 18.4	0.079	-	29 (15–44)
No anemia *	10	137.5 ± 15.5	141.5 ± 11.8	0.596	30%	29 (17–42)
Mild anemia *	10	114.2 ± 4.4	116.4 ± 11.9	0.586	10%	20.5 (15–29)
Moderate and severe anemia *	25	94.7 ± 2	105.4 ± 3.2	0.004	28%	31 (14–44)
Platelet count (×10^9^/L)	45	240.6 ± 114.6	238.9 ± 92.3	0.942	-	29 (15–44)
>150 × 10^9^/L	33	290.7 ± 15.6	252 ± 20.7	0.134	30.3%	29 (17–42)
<150 × 10^9^/L	12	102.7 ± 8.3	196.5 ± 17.5	0.001	8.3%	37 (9–100)
Leucocytes(×10^9^/L)	45	6.42	6.51	0.887	-	29 (15–44)

* In accordance with definition in Section 4.2. No anemia was considered when; Hb > 130 g/L for men and Hb > 120 g/L for women. Mild anemia; Hb 110–129 g/L for men and Hb 110–119 g/L for women. Moderate anemia considered Hb < 109 g/L and severe anemia Hb < 80 g/L for men and women in both cases.

## Data Availability

The data presented in this study are available on request from the corresponding author (omurillo@bellvitgehospital.cat).

## References

[1] Zhanel G.G., Love R., Adam H., Golden A., Zelenitsky S., Schweizer F., Gorityala B., Lagacé-Wiens P.R.S., Rubinstein E., Walkty A. (2015). Tedizolid: A novel oxazolidinone with potent activity against multidrug-resistant gram-positive pathogens. Drugs.

[2] Flanagan S., Fang E., Muñoz K.A., Minassian S.L., Prokocimer P.G. (2014). Single- and multiple-dose pharmacokinetics and absolute bioavailability of tedizolid. Pharmacotherapy.

[3] Douros A., Grabowski K., Stahlmann R. (2015). Drug-drug interactions and safety of linezolid, tedizolid, and other oxazolidinones. Expert Opin. Drug Metab. Toxicol..

[4] Prokocimer P., De Anda C., Fang E., Mehra P., Das A. (2013). Tedizolid phosphate vs linezolid for treatment of acute bacterial skin and skin structure infections: The ESTABLISH-1 randomized trial. JAMA.

[5] Moran G.J., Fang E., Corey G.R., Das A.F., De Anda C., Prokocimer P. (2014). Tedizolid for 6 days versus linezolid for 10 days for acute bacterial skin and skin-structure infections (ESTABLISH-2): A randomised, double-blind, phase 3, non-inferiority trial. Lancet Infect. Dis..

[6] Morata L., Tornero E., Martínez-Pastor J.C., García-Ramiro S., Mensa J., Soriano A. (2014). Clinical experience with linezolid for the treatment of orthopaedic implant infections. J. Antimicrob. Chemother..

[7] Senneville E., Legout L., Valette M., Yazdanpanah Y., Beltrand E., Caillaux M., Migaud H., Mouton Y. (2006). Effectiveness and tolerability of prolonged linezolid treatment for chronic osteomyelitis: A retrospective study. Clin. Ther..

[8] Cobo J., Lora-Tamayo J., Euba G., Jover-Sáenz A., Palomino J., Del Toro M.D., Rodríguez-Pardo D., Riera M., Ariza J. (2013). Linezolid in late-chronic prosthetic joint infection caused by gram-positive bacteria. Diagn. Microbiol. Infect. Dis..

[9] Boak L.M., Rayner C.R., Grayson M.L., Paterson D.L., Spelman D., Khumra S., Capitano B., Forrest A., Li J., Nation R.L. (2014). Clinical population pharmacokinetics and toxicodynamics of linezolid. Antimicrob. Agents Chemother..

[10] Blassmann U., Roehr A.C., Frey O.R., Koeberer A., Briegel J., Huge V., Vetter-Kerkhoff C. (2016). Decreased Linezolid Serum Concentrations in Three Critically Ill Patients: Clinical Case Studies of a Potential Drug Interaction between Linezolid and Rifampicin. Pharmacology.

[11] Pea F., Viale P., Cojutti P., Del pin B., Zamparini E., Furlanut M. (2012). Therapeutic drug monitoring may improve safety outcomes of long-term treatment with linezolid in adult patients. J. Antimicrob. Chemother..

[12] Legout L., Valette M., Dezeque H., Nguyen S., Lemaire X., Loïez C., Caillaux M., Beltrand E., Dubreuil L., Yazdanpanah Y. (2010). Tolerability of prolonged linezolid therapy in bone and joint infection: Protective effect of rifampicin on the occurrence of anaemia?. J. Antimicrob. Chemother..

[13] Gandelman K., Zhu T., Fahmi O.A., Glue P., Lian K., Obach R.S., Damle B. (2011). Unexpected effect of rifampin on the pharmacokinetics of linezolid: In silico and in vitro approaches to explain its mechanism. J. Clin. Pharmacol..

[14] Morales-Molina J.A., de Antonio J.M., Marín-Casino M., Grau S. (2005). Linezolid-associated serotonin syndrome: What we can learn from cases reported so far. J. Antimicrob. Chemother..

[15] Taylor J.J., Wilson J.W., Estes L.L. (2006). Linezolid and serotonergic drug interactions: A retrospective survey. Clin. Infect. Dis..

[16] Lodise T.P., Bidell M.R., Flanagan S.D., Zasowski E.J., Minassian S.L., Prokocimer P. (2016). Characterization of the haematological profile of 21 days of tedizolid in healthy subjects. J. Antimicrob. Chemother..

[17] Park K.-H., Greenwood-Quaintance K.E., Schuetz A.N., Mandrekar J.N., Patel R. (2017). Activity of Tedizolid in Methicillin-Resistant Staphylococcus epidermidis Experimental Foreign Body-Associated Osteomyelitis. Antimicrob. Agents Chemother..

[18] Park K.-H., Greenwood-Quaintance K.E., Mandrekar J., Patel R. (2016). Activity of Tedizolid in Methicillin-Resistant Staphylococcus aureus Experimental Foreign Body-Associated Osteomyelitis. Antimicrob. Agents Chemother..

[19] Carvalhaes C.G., Sader H.S., Flamm R.K., Mendes R.E. (2019). Tedizolid in vitro activity against Gram-positive clinical isolates causing bone and joint infections in hospitals in the USA and Europe (2014–17). J. Antimicrob. Chemother..

[20] Si S., Durkin M.J., Mercier M.M., Yarbrough M.L., Liang S.Y. (2017). Successful treatment of prosthetic joint infection due to vancomycin-resistant enterococci with tedizolid. Infect. Dis. Clin. Pract..

[21] Kullar R., Puzniak L.A., Swindle J.P., Lodise T. (2020). Retrospective Real-World Evaluation of Outcomes in Patients with Skin and Soft Structure Infections Treated with Tedizolid in an Outpatient Setting. Infect. Dis. Ther..

[22] Vendrell M.M., Pitarch M.T., Lletí M.S., Muñoz E.C., Ruiz L.M., Lao G.C., Suñé E.L., Pueyo J.M., Sempere M.R.O., Montoya M.L.P.B. (2020). Safety and tolerability of more than six days of tedizolid treatment. Antimicrob. Agents Chemother..

[23] Gerson S.L., Kaplan S.L., Bruss J.B., Le V., Arellano F.M., Hafkin B., Kuter D.J. (2002). Hematologic effects of linezolid: Summary of clinical experience. Antimicrob. Agents Chemother..

[24] Flanagan S., McKee E.E., Das D., Tulkens P.M., Hosako H., Fiedler-Kelly J., Passarell J., Radovsky A., Prokocimer P. (2015). Nonclinical and pharmacokinetic assessments to evaluate the potential of tedizolid and linezolid to affect mitochondrial function. Antimicrob. Agents Chemother..

[25] Ferry T., Batailler C., Conrad A., Triffault-Fillit C., Laurent F., Valour F., Chidiac C., Ferry T., Valour F., Perpoint T. (2018). Correction of Linezolid-Induced Myelotoxicity After Switch to Tedizolid in a Patient Requiring Suppressive Antimicrobial Therapy for Multidrug-Resistant Staphylococcus epidermidis Prosthetic-Joint Infection. Open Forum Infect. Dis..

[26] Egle H., Trittler R., Kümmerer K., Lemmen S. (2005). Linezolid and rifampin: Drug interaction contrary to expectations?. Clin. Pharmacol. Ther..

[27] Flanagan S., Bartizal K., Minassian S.L., Fang E., Prokocimer P. (2013). In vitro, In Vivo, and clinical studies of tedizolid to assess the potential for peripheral or central monoamine oxidase interactions. Antimicrob. Agents Chemother..

[28] Osmon D.R., Berbari E.F., Berendt A.R., Lew D., Zimmerli W., Steckelberg J.M., Rao N., Hanssen A., Wilson W.R. (2013). Diagnosis and Management of Prosthetic Joint Infection: Clinical Practice Guidelines by the Infectious Diseases Society of America. Clin. Infect. Dis..

[29] Lew D.P., Waldvogel F.A. (2004). Osteomyelitis. Lancet.

[30] Lipsky B.A., Senneville É., Abbas Z.G., Aragón-Sánchez J., Diggle M., Embil J.M., Kono S., Lavery L.A., Malone M., Asten S.A. (2020). Guidelines on the diagnosis and treatment of foot infection in persons with diabetes (IWGDF 2019 update). Diabetes Metab. Res. Rev..

